# Recombinant Microneme Proteins MIC1 and MIC4 from *Toxoplasma gondii* Cause Cytotoxic Effects in the Human Jurkat T-Lymphocyte Cell Line

**DOI:** 10.3390/pathogens14040372

**Published:** 2025-04-09

**Authors:** Igor E. L. Souza, Maria-Cristina Roque-Barreira, Ademilson Panunto-Castelo

**Affiliations:** 1 Department of Cell and Molecular Biology and Pathogenic Bioagents, Ribeirão Preto Medical School, University of São Paulo, Ribeirão Preto 14040-901, SP, Brazil; igor.elemosdesouza@gmail.com (I.E.L.S.); mcrbarre@fmrp.usp.br (M.-C.R.-B.); 2 Department of Biology, Faculty of Philosophy, Sciences and Letters at Ribeirão Preto, Ribeirão Preto 14040-901, SP, Brazil

**Keywords:** *Toxoplasma gondii*, microneme, lectins, apoptosis, Jurkat cells

## Abstract

*Toxoplasma gondii* is an obligate intracellular parasite that causes toxoplasmosis, a potentially devastating disease to fetuses and immunocompromised individuals. Among its microneme proteins, MIC1 and MIC4 play crucial roles in host-parasite interactions, facilitating adhesion by binding glycans on host cells. Beyond these roles, these lectins have been implicated in modulating immune responses and inducing apoptosis, but their effects on human immune cells remain unclear. Here, we investigated the interaction of recombinant MIC1 (rMIC1) and rMIC4 with Jurkat T lymphocytes, a human immune cell model. Both lectins bound Jurkat cells in a carbohydrate-dependent manner, with rMIC4 showing competitive binding over rMIC1. Importantly, we observed that rMIC1 and rMIC4 reduced Jurkat cell viability in a time- and dose-dependent manner, inducing apoptosis through caspase activation by extrinsic and intrinsic pathways. The apoptosis was driven by reactive oxygen species production via the NADPH oxidase complex and the activation of p38 and JNK MAPK signaling pathways, emphasizing the ability of these lectins to modulate cellular signaling cascades. This study offers insights into the mechanisms involved in MIC1 and MIC4 interactions with immune cells.

## 1. Introduction

Toxoplasmosis is an infection caused by *Toxoplasma gondii*, an obligate intracellular parasite from the phylum Apicomplexa. Even though toxoplasmosis is usually asymptomatic or produces mild symptoms, it can cause severe disseminated disease and even death in fetuses and immunodeficient patients [1]. Human infection typically occurs through the ingestion of tissue cysts in raw or undercooked meat or oocysts present in contaminated water, food, or soil [1,2]. Toxoplasmosis represents a global health problem, with an incidence rate of approximately 1.5 cases of congenital toxoplasmosis per 1000 live births and a disease burden of 1.2 million disability-adjusted life years [3].

Cell infection by *T. gondii* is a multistep process involving attachment, penetration, and internalization, which depends on the contents of specialized organelles, namely micronemes, rhoptries, and dense granules [4,5]. Micronemes are rod-shaped secretory organelles located in the anterior part of the parasite cell that release essential proteins to the adhesion of the parasite to the host cell in the initial stages of invasion [5,6]. Most microneme proteins contain modular structures that are homologous to adhesion or the binding domains present in higher eukaryotes, such as the epidermal growth factor-like domain, chitin-binding-like domain, and the thrombospondin-like repeats [7]. One of the complexes exposed on the parasite surface after microneme discharge comprises MIC1, MIC4, and MIC6 [8]. MIC6 is an integral membrane protein that binds and escorts the complex [9], while MIC1 and MIC4 have carbohydrate-recognition domains (CRDs) that interact with sialyl and galactosyl terminals, respectively, in the surface glycans of host cells [10,11,12,13].

In addition to functioning as an adhesin to the parasite-host attachment, MIC1 and MIC4 interact and activate immune cells. When adherent mouse spleen cells were treated with a lactose-binding fraction (Lac^+^) of soluble *T. gondii* antigens containing MIC1 and MIC4, they produced seven times more interleukin (IL-) 12 than the non-treated cells. We supposed that MIC1 and MIC4 act as a pathogen-associated molecular pattern (PAMP) because they bind to two pattern recognition receptors (PRRs), namely Toll-like receptor (TLR) 2 and TLR4, on mouse macrophages and dendritic cells and upregulate IL-12 production [14]. The activity of MIC1 was dependent on their interaction with the second, third, and fourth N-glycans on the TLR2, whereas MIC4 exclusively targets the third N-glycan of the TLR2. Moreover, the interaction between MIC1 or MIC4 and TLR2 can initiate the production of pro-inflammatory cytokines, which is enhanced by the co-receptor CD14 and the heterodimerization of TLR2 with TLR1 or TLR6. In addition to triggering the production of pro-inflammatory cytokines, such as IL-12, IL-6, and TNF, MIC1 and MIC4 also stimulate the release of the anti-inflammatory cytokine IL-10 [14,15], probably regulating the immune response [16].

After infection by *T. gondii*, IL-12 from mononuclear phagocytes prompts NK and CD4^+^ T cells to release IFN-γ, driving the immune response towards a Th1 pattern [17,18,19]. This response is crucial to the development of asymptomatic chronic infection, and MIC1 and MIC4 might contribute to this outcome, since the Lac^+^ fraction, along with recombinant microneme proteins (rMIC) 1 and rMIC4, provide protective immunity against *T. gondii* infection in mice [20,21].

Lectins are crucial in many biological processes, such as the trafficking, targeting, and clearance of proteins and cell adhesion, immunity, infection, and apoptosis [22]. The latter was first studied when rats were injected with the toxic lectins ricin and abrin, developing apoptotic-like bodies abundant in para-aortic lymph nodes, Peyer’s patches, and ileal crypts [23]. After that, many other studies showed apoptosis induction in immune and cancer cells [24,25]. In addition to apoptosis, lectins can participate in different types of cell death, such as autophagy, necroptosis, and pyroptosis [26]. These processes may result from receptor blocking, apoptotic receptor cross-linking, growth factor interference, lysosomal destabilization, protein synthesis inhibition, or reactive oxygen species (ROS) production [27].

In this study, we aim to evaluate the effects of *T. gondii* rMIC1 and rMIC4 on Jurkat cells, a neoplastic human T-cell line widely used as a model for human immune cells. Specifically, we assessed their binding to the cell surface and their impact on cell survival. Furthermore, we investigated the signaling cascades activated by rMIC1 and rMIC4 and analyzed the relative contributions of various signaling pathways to their observed effects on Jurkat T cells.

## 2. Materials and Methods

### 2.1. Jurkat Cell Cultures

Jurkat E6 cells (ATCC: TIB-152) were cultured in an RPMI 1640 medium supplemented with 4.5 g/L glucose (Sigma-Aldrich, St. Louis, MO, USA), 100 U/mL of penicillin, and 100 µg/mL of streptomycin and 10% fetal bovine serum (Thermo Fisher Scientific, Waltham, MA, USA) at 37 °C in a humidified 5% CO_2_ atmosphere. The culture medium was changed every 2 or 3 days. Only cultures with cell viability above 95% were used in the experiments.

### 2.2. Recombinant Proteins

Endotoxin-free recombinant MIC1 and MIC4 were produced and purified as described by Mendonça-Natividade et al. [15]. Briefly, endotoxin-free recombinant MIC1 and MIC4 were produced and purified using *E. coli* BL21 (DE3) transformed with pDEST17-MIC1 or pDEST17-MIC4. Bacterial cultures were grown in an LB medium supplemented with ampicillin (100 μg/mL) (LBamp) at 37 °C in an orbital shaker at 220 rpm. A 1% volume of freshly grown pre-inoculum was transferred into 500 mL of LBamp and grown until it reached an optical density at 600 nm of 0.4–0.6. Protein expression was induced with 0.5 mM isopropyl-β-D-thiogalactopyranoside (IPTG) (Sigma-Aldrich). After induction for 4 h, cells were harvested by centrifugation, and pellets were resuspended in a Tris-HCl buffer (pH 7.5) containing 100 mM NaCl, 5 mM EDTA, 0.1 mM PMSF, 4 μL DNase (20,000 U), and lysozyme at 1 mg/mL. Lysis was performed by sonication, followed by centrifugation at 15,200× *g* for 15 min at 4 °C to collect the pellet containing the inclusion bodies. The pellet proteins were solubilized in a 100 mM Tris–HCl buffer (pH 7.4) containing 8 M urea and 50 mM glycine overnight with stirring at room temperature. Solubilized inclusion bodies were centrifuged at 15,200× *g* for 40 min at 4 °C. The supernatant was subjected to stepwise dialysis against 100 mM Tris-HCl buffer (pH 7.4) with decreasing urea concentrations to facilitate refolding. Endotoxin removal was achieved using polymyxin B columns (Bio-Rad Laboratories, Hercules, CA, USA), ensuring endotoxin levels below 0.1 EU/mL. Finally, the biotinylation of recombinant MIC1 and MIC4 was performed using EZ-Link Sulfo-NHS-LC-Biotin (Thermo Fisher Scientific), following the manufacturer’s recommendations, for further functional studies.

### 2.3. Binding Assay

Cell suspensions of 5 × 10^5^ cells/mL of the Jurkat strain were fixed at room temperature with 4% paraformaldehyde in PBS for 30 min. Then, the cells were washed in PBS, and the binding was performed with different concentrations of biotinylated rMIC1 or rMIC4 at temperatures of 4 °C, 25 °C, or 37 °C. After 2 h, the cells were washed and incubated with 5 µg/mL of streptavidin-FITC for 40 min. After two consecutive washes with PBS, the cells were acquired on a Guava EasyCyte Mini System (Merck Millipore, Billerica, MA, USA). A minimum of 5000 events were acquired. All data analyses were performed using FlowJo software version 10 (BD Biosciences). The gates were set based on forward and side scatter parameters to exclude debris and dead cells (Figure S1, panel A). The analysis was performed on viable cells, and the FITC fluorescence intensity was measured in the FL1 channel, representing the binding of biotinylated rMIC1 or rMIC4. In some experiments, fetuin and asialofetuin were added simultaneously to biotinylated rMIC1 or rMIC4 to block their CRDs. In others, Jurkat cells were treated with 6 U/mL of neuraminidase (Sigma-Aldrich) in acetate buffer, with pH 5.5, or 50 U/mL β-galactosidase (Sigma-Aldrich) in citrate buffer, with pH 6.0, for 1 h at 37 °C. After digestion, the cells were washed and labeled with biotinylated rMIC1 or rMIC4. The cytometry data acquisition and analysis were conducted as described above.

### 2.4. Evaluation of Jurkat Cells Viability After Stimulus with rMIC1 or rMIC4

Cultures of 2.5 × 10^5^ Jurkat cells/mL were stimulated with different concentrations of rMIC1 or rMIC4 for 24 or 48 h at 37 °C in a humidified 5% CO_2_ atmosphere. In additional experiments, cultures of 3 × 10^4^ Jurkat cells/mL were stimulated with different concentrations of rMIC1 or rMIC4 for 7 days at 37 °C. In some cultures, the cells were washed one hour after the stimulation with rMIC1 and rMIC4 and cultured for 7 days. In competition assays, 2.5 × 10^5^ Jurkat cells/mL were stimulated with 5 μg/mL of rMIC1 or rMIC4 alongside different concentrations of fetuin or asialofetuin for 24 h. Four hours before the end of the experiments, 5 µL of a 3-(4,5-dimethyl-2-thiazolyl)-2,5-diphenyl-2H-tetrazolium bromide (MTT, Sigma-Aldrich) solution at 5 mg/mL were added to the cell culture. MTT was used to measure mitochondrial activity, and the percentage of cell viability and growth inhibition was determined by reading the absorbance at 570 nm of the cell lysate, where formazan accumulates as a product of MTT reduction. The plates were centrifuged at 300× *g* for 10 min at room temperature, and after culture supernatant removal, 100 µL of dimethyl sulfoxide (DMSO, Sigma-Aldrich) were added to each well. The plates were protected from light, shaken for 10 min, and then read at 570 nm in a PowerWave-X spectrophotometer (BioTek Instruments, Winooski, VT, USA).

The cell viability was calculated as a percentage control (unstimulated cells): cell viability (%) = [(optical density value from the rMIC1- or rMIC4-stimulated cell lysate/average of optical density value from unstimulated cell lysates) × 100. The inhibition percentage of cell growth was calculated as follows: inhibition of cell growth (%) = [1-(optical density value from the rMIC1- or rMIC4-stimulated cell lysates/average of optical density value from unstimulated cells lysates)] × 100.

### 2.5. Apoptosis Detection

Jurkat cells at the density of 2.5 × 10^5^ cells/mL were stimulated with different concentrations of rMIC1 or rMIC4. After 24 h of incubation at 37 °C in a humidified 5% CO_2_ atmosphere, the cell suspensions were stained with BD Pharmingen FITC Annexin V Apoptosis Detection Kit I (BD Bioscience, Franklin Lakes, NJ, USA), following the manufacturer’s protocols. The cells were acquired on a FACS Canto flow cytometer (BD Bioscience). A minimum of 5000 events were acquired. Data analyses were performed using FlowJo X software (BD Biosciences). Cells were gated based on forward and side scatter parameters to exclude debris, then analyzed by labeling with Annexin V-FITC in the FL1 channel to detect early apoptosis and propidium iodide (PI) in the FL3 channel to identify late apoptosis or necrosis (Figure S1, panel B). To confirm the apoptotic effect of rMIC1 and rMIC4, we incubated the cultures with 15 µM of Emricasan, a pan-caspase inhibitor, or DMSO (Sigma-Aldrich) as control. Cell suspensions were stained with 5 µg/mL of PI for 10 min, followed by cytometric data acquisition and analysis, as described above in Figure S1, panel C.

### 2.6. Caspase Activity Evaluation

Jurkat cells at 2.5 × 10^5^ cells/mL were stimulated with 5 µg/mL of rMIC1 or rMIC4 at 37 °C in a humidified 5% CO_2_ atmosphere for 24 h. As a positive control, we used 10 µg/mL of As_2_O_3_ (Sigma-Aldrich) to active caspases-3/7 and 15 µg/mL of cycloheximide (Sigma-Aldrich) to active caspase-8. To determine the activation of initiator caspases in the extrinsic pathway (caspase-8) and the executioner caspase- 3 and -7, we used Vybrant FAM Caspase-8 Assay Kit and Vybrant FAM Caspase-3 and -7 Assay Kit (Thermo Fisher Scientific), respectively, following the manufacturer’s instructions. Cell suspensions were acquired on the Guava EasyCyte Mini System (Merck Millipore) and analyzed with FlowJo X software (BD Biosciences), following the manufacturer’s instructions for the kits. The gate strategy for caspase-8 detection was performed as indicated in Figure S1, panel C.

### 2.7. Assessment of the Mitochondrial Membrane Potential

Jurkat cells at a density of 2.5 × 10^5^ cells/mL were stimulated with 5 µg/mL of rMIC1 or rMIC4. After 24 h of incubation, the cells were washed twice and stained with DiOC6 the 3,3′-dihexyloxacarbocyanine iodide [DiOC6(3), Thermo Fisher Sicentific] to a final concentration of 50 nM, for 10 min, at 37 °C. Then, the cells were washed with PBS, centrifuged at 300× *g*, and resuspended with an RPMI medium. The cells were washed twice in PBS, acquired on the Guava EasyCyte Mini System (Merck Millipore), and analyzed with FlowJo X software (BD Biosciences). The gates were set based on forward and side scatter parameters to exclude debris and dead cells. The analysis was performed on viable cells, and the FITC fluorescence intensity was measured in the FL1 channel, representing the mitochondrial membrane potential (Figure S1, panel D).

### 2.8. Chromatin Fragmentation Analysis

Jurkat cell cultures at 5 × 10^6^ cells/mL were stimulated with 5 µg/mL of rMIC1 or rMIC4. PBS and arsenic trioxide (As_2_O_3_) at 10 µg/mL were used as negative and positive controls, respectively. After 16 h, the cells were collected, washed in ice-cold PBS, and centrifuged at 300× *g* for 10 min at 4 °C. The cell pellet was disrupted with 200 µL of lysis buffer (100 mM NaCl, 20 mM Tris, 10 mM EDTA, 0.5% SDS, and 1% Triton X-100, pH 8.0). Then, 1 µg/mL of RNase (Sigma-Aldrich) was added, and the cell lysate was left in a dry bath for 1 h at 37 °C. Afterward, 10 µg/mL of proteinase K (Thermo Fisher Scientific) was added, following incubation for 16 h at 56 °C. The lysate was run on a 1.5% agarose gel containing a 0.1% SYBR Safe DNA gel stain (Thermo Fisher Scientific). The images of electrophoresis gels were acquired by a ChemiDoc XRS+ System (Bio-Rad Laboratories) using Image Lab software version 5.2 (Bio-Rad Laboratories).

### 2.9. Identification of Cell Signaling Pathways Used by MICs to Cause Cell Death

To identify the mechanisms of cell death caused by rMIC1 and rMIC4, we used pharmacological inhibitors of MAPKs and phosphoinositide 3-kinase (PI3K). PD98059 at 5 µM was used to inhibit the extracellular signal-regulated protein kinases (ERKs), JNK-IN-8 at 3 µM to C-Jun N-terminal kinases (JNK), SB239063 at 10 µM to p38 MAPK, and LY294002 at 5 µM to PI3K. All chemical inhibitors were purchased from Sigma-Aldrich. To ensure the block of MAPKs and PI3K, cultures containing 2 × 10^5^ Jurkat cells/mL were incubated with the inhibitors for 1 h. DMSO (Sigma-Aldrich) was used as a negative control. Then, the Jurkat cells were incubated with 5 µg/mL of rMIC1 or rMIC4 and cultured at 37 °C in a humidified 5% CO_2_ atmosphere for 24 h. The percentage of viable cells after inhibition of ERK and PI3K pathways was determined by the MTT protocol as described above. The analysis of inhibition of JNK and p38 was seen by PI labeling, following acquisition on the Guava EasyCyte Mini System (Merck Millipore), and the analysis was conducted as described above.

### 2.10. Detection of ROS

Cultures of 2 × 10^5^ Jurkat cells/mL were labeled with the 2′,7′-dichlorofluorescin diacetate (DCFDA) (Sigma-Aldrich) probe at a concentration of 5 μM. After 45 min, the cells were washed in PBS, centrifuged at 300× *g*, and resuspended in RPMI protected from light. Cells were plated and stimulated for 2 h with different concentrations of rMIC1 or rMIC4 at 37 °C in a humidified 5% CO_2_ atmosphere. Then, the plates were read in the BioTek FLx800 TB Microplate Fluorescence Reader (BioTek Instruments). Similar experiments to detect ROS inhibition were conducted by incubation of the Jurkat cells with an inhibitor of NADPH oxidase complex, the diphenylene iodonium (DPI) at 20 μM, and the inhibitors of mitochondrial ROS, the mito-TEMPO (MIT) at 20 μM and the N-acetyl-cysteine (NAC) at 1 mM. All inhibitors were purchased from Sigma-Aldrich.

### 2.11. Statistical Analysis

Data are presented as mean ± standard deviation. Statistical analysis was performed using one-way ANOVA followed by Bonferroni post hoc testing, with statistical significance set at a *p*-value of less than 0.05. All analyses were conducted using GraphPad Prism 8.0 software (GraphPad Software, Inc., La Jolla, CA, USA).

## 3. Results

### 3.1. rMIC1 and rMIC4 Interact with Glycans on Jurkat Cells

**Figure 1:**
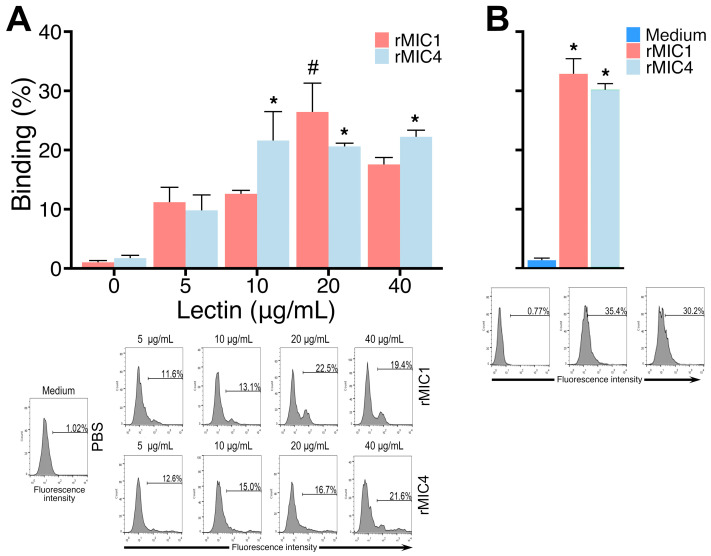
Evaluation of MIC1 and MIC4 binding to glycans on Jurkat cells. (**A**) Jurkat cells (5 × 10^5^ cells) were fixed, incubated with varying concentrations of biotinylated lectin rMIC1 or rMIC4 at 4 °C for 2 h, and labeled with streptavidin-FITC before cytometry analysis. (**B**) Similar experiments with these lectins at 20 μg/mL were performed at 37 °C. Results, expressed as mean ± SD of triplicates from a representative experiment repeated three times with similar outcomes, are shown in histograms representing one triplicate. * *p* < 0.05 compared to the groups incubated without the lectins (0 in panel (**A**) or medium in panel (**B**)) or with 5 μg/mL of rMIC4 (in panel **A**). # *p* < 0.05 compared to the groups incubated with other rMIC1 concentrations.

Although the molecules involved in the invasion process of mouse host cells by *T. gondii* are well-known, they have been studied less in the context of this process in human cells. Here, we evaluated the interactions of recombinant *T. gondii* lectins, MIC1 and MIC4, with a human T-lymphocyte cell line, the Jurkat cells. First, we assessed the optimal concentration of rMIC1 and rMIC4 for binding assays on Jurkat cells at 4 °C. We found that rMIC1 at 20 μg/mL exhibited better binding to Jurkat cells than other concentrations of this lectin. For rMIC4, 10 μg/mL demonstrated higher cell binding than 5 μg/mL, while no significant difference was observed among concentrations of 10, 20, and 40 μg/mL (Figure 1, panel A). We also tested rMIC1 and rMIC4 at 20 μg/mL at 37 °C and observed a binding slightly higher than at 4 °C (Figure 1, panel B).

**Figure 2:**
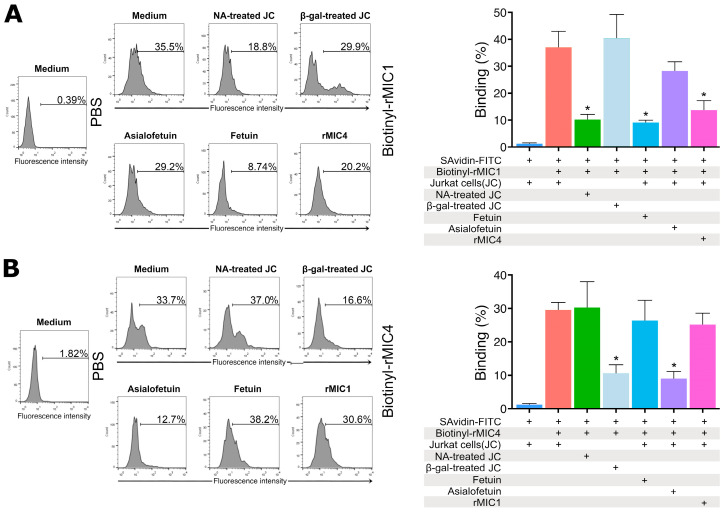
Binding of rMIC1 and rMIC4 to Jurkat cells. Fixed Jurkat cells (5 × 10^5^ cells) were incubated with 30 μg/mL of biotinylated rMIC1 (**A**) or rMIC4 (**B**) as positive controls. Neuraminidase- or β-galactosidase-treated cells (NA- or β-gal-treated JC) were used in some assays. For competition, rMIC1 was incubated with 400 μg/mL of fetuin, and rMIC4 was incubated with the same amount of asialofetuin. Cross-competition involved biotinylated rMIC1 with unlabeled rMIC4 (**A**) or biotinylated rMIC4 with unlabeled rMIC1 (**B**). Negative controls included cells treated with streptavidin-FITC alone (SAvidin-FITC). Samples were acquired on a Guava EasyCyte Mini cytometer, and the data were analyzed using the FlowJo program. Results, expressed as mean ± SD of triplicates from a representative experiment repeated three times with similar outcomes, are shown in histograms representing one triplicate. * *p* < 0.05 compared to positive and negative controls. The bar colors represent different sets of reaction conditions.

Next, we analyzed whether the binding of rMIC1 and rMIC4 on Jurkat cells was dependent on carbohydrates. For this, we incubated rMIC1 or rMIC4 simultaneously with a highly glycosylated protein, fetuin, and Jurkat cells, and we found a significant decrease in the binding of rMIC1 to these cells (Figure 2, panel A), but not of rMIC4 (Figure 2, panel B). Similarly, when we incubated rMIC1 or rMIC4 with neuraminidase-treated Jurkat cells, only rMIC1 had a significantly lower binding when compared with non-treated cells (Figure 2, panels A and B). In similar experiments using desialylated fetuin (asialofetuin) as a competitor or β-galactosidase-treated Jurkat cells, only rMIC4 showed significantly lower binding to Jurkat cells when compared with non-treated cells (Figure 2, panel B). As expected, these results suggested that the interaction of MIC1 with Jurkat cell surface glycans depends on sialic acid, whereas rMIC4 is on β-galactopyranoside.

Since MIC1 and MIC4 are relevant adhesins for cell invasion by *T. gondii*, we wondered whether they would synergistically affect each other. Surprisingly, when we incubated simultaneously labeled rMIC1 and unlabeled rMIC4 with Jurkat cells, there was a considerable reduction in the interaction of rMIC1 with the cell surface (Figure 2, panel A), suggesting that MIC4 has an antagonistic effect on MIC1 as regards the binding to the cells’ surface. However, the opposite was not seen—incubating labeled rMIC4 and unlabeled rMIC1 with Jurkat cells did not affect rMIC4 binding (Figure 2, panel B). These results suggest that rMIC4 interferes with the interaction of MIC1 with Jurkat cells.

### 3.2. rMIC1 and rMIC4 Induce Time- and Dose-Dependent Loss of Viability and Growth Inhibition in Jurkat Cells

**Figure 3:**
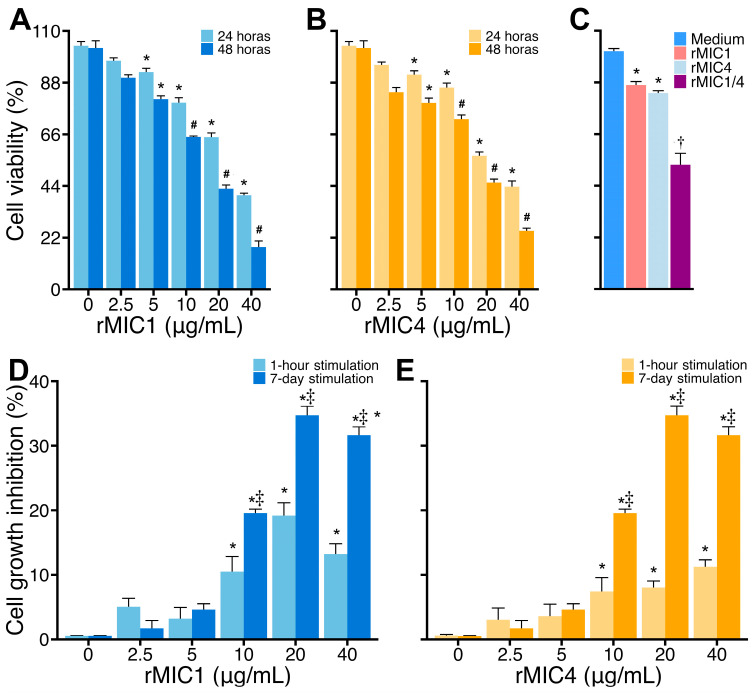
rMIC1 and rMIC4 reduce Jurkat cell viability in extended incubation. Jurkat cells (2 × 10^5^ cells/mL) were incubated with increasing concentrations of rMIC1 (**A**) or rMIC4 (**B**) for 24 or 48 h. (**C**) For 24-h cultures, cells were treated with 5 µg/mL rMIC1, rMIC4, or both. Seven-day cultures (3 × 10^4^ Jurkat cells/mL) were continuously treated with different concentrations of rMIC1 (**D**) or rMIC4 (**E**) or stimulated for 1 h, followed by washing, resuspension with a fresh medium, and maintenance for 7 days. Unstimulated cells were used as the negative control. Cell viability was assessed by MTT assay, and the absorbance was measured at 570 nm. The results are expressed as mean ± SD of triplicates from a single representative experiment repeated three times with similar outcomes. * *p* < 0.05 compared to non-stimulated cells (0). # *p* < 0.05 compared to the group incubated with same concentration of rMIC1 (**A**) or rMIC4 (**B**) for 24 h. † *p* < 0.05 compared to other groups. ‡ *p* < 0.05 compared to the group stimulated with rMIC1 or rMIC4 for 1-h stimulation followed by 7-day culturing.

Previous studies have shown that lectins can alter the proliferation pattern of Jurkat cells (reviewed by [28]), leading us to hypothesize that rMIC1 and rMIC4 might have a similar effect). Thus, we incubated Jurkat cells with different concentrations of rMIC1 or rMIC4 for 24 and 48 h, and we noticed that both rMIC1 and rMIC4 induced the cell viability loss in a time- and dose-dependent manner (Figure 3, panels A and B). Given the modulatory effect of rMIC4 on the interaction of rMIC1 with cell surface glycans, we also evaluated whether the simultaneous incubation of rMIC1 and rMIC4 with Jurkat cells would alter the cytotoxic effect compared to exposure to only one of the lectins. In contrast to the binding assays, the simultaneous incubation of rMIC1 and rMIC4 led to a synergistic effect, significantly decreasing cell viability more than when the cells were treated with just one of the lectins (Figure 3, panel C).

The viability loss of Jurkat cells induced by MIC1 and MIC4 for 48 h led us to investigate the influence of these lectins over a longer culture time. When Jurkat cells were cultured for seven consecutive days (7-day stimulation) in the presence of various concentrations of rMIC1 or rMIC4, both lectins reduced cell growth in a concentration-dependent manner, with effects starting at 10 μg/mL (Figure 3, panels D and E). Next, we wondered if the stimulus for only 1 h with different concentrations of rMIC1 and rMIC4 in the cultures lasting 7 days would also reduce cell growth. While a 1-h stimulation with the lectins was sufficient to induce some loss of cell viability, the effect was significantly less pronounced than with continuous stimulation (Figure 3, panels D and E).

**Figure 4:**
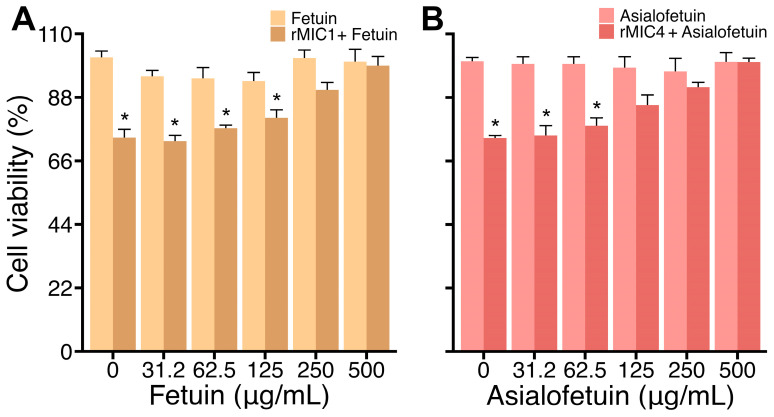
Jurkat cell viability loss after stimulation with rMIC1 or rMIC4 is dependent upon carbohydrate recognition. Jurkat cells (2 × 10^5^ cells/mL) were treated with 5 µg/mL of rMIC1 (**A**) or rMIC4 (**B**) and varying concentrations of fetuin (**A**) or asialofetuin (**B**) for 24 h. Cells treated with only fetuin or asialofetuin were used as the negative controls. MTT assays measured viability, expressed as mean ± SD of triplicates from one representative experiment repeated three times with similar outcomes. * *p* < 0.05 compared to non-stimulated cells (negative controls).

Lectins can induce cytotoxicity and usually do so by recognizing glycosylated receptors on the surface of cells [25]. Therefore, we analyzed whether the loss of viability triggered by rMIC1 and rMIC4 depended on carbohydrate recognition by incubating Jurkat cell cultures with rMIC1 or rMIC4 in the presence of fetuin, a heavily glycosylated protein, or its desialylated form, asialofetuin. We observed that rMIC1 activity was blocked with fetuin as a competitor starting at 250 µg/mL (Figure 4, panel A), whereas the rMIC4 one was reversed with asialofetuin starting at 125 µg/mL (Figure 4, panel B). These data suggest that rMIC1 and rMIC4 induce the loss of Jurkat cell viability through carbohydrate recognition.

### 3.3. rMIC1 and rMIC4 Induce Apoptosis in Jurkat Cells

**Figure 5:**
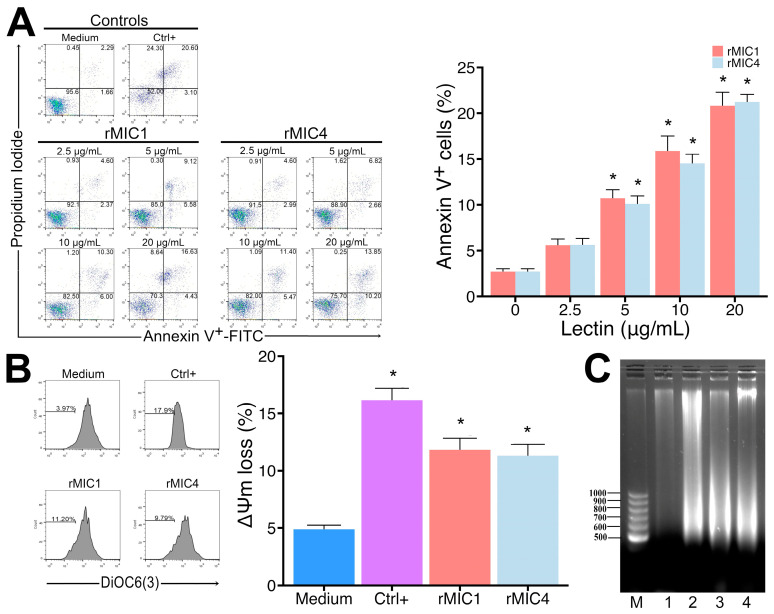
rMIC1 and rMIC4 induce apoptosis in Jurkat cells. (**A**) Jurkat cells (2.5 × 10^5^ cells/mL) were treated with rMIC1 or rMIC4 for 24 h at varying concentrations and stained with annexin V-FITC and PI. Medium and arsenic trioxide (As_2_O_3_) at 10 µg/mL were used as negative and positive controls, respectively. Samples were acquired using a cytometer, and data were analyzed using the FlowJo program. The bar graph on the right side of the figure represents the results of early and late apoptosis, i.e., annexin V single-positive cells and annexin V/PI double-positive cells. (**B**) Jurkat cells (3 × 10^5^ cells/mL) were treated with 5 µg/mL rMIC1 or rMIC4, stained with 50 nM DiOC6(3) for mitochondrial membrane potential (Δψm), and analyzed as described above. The number indicated in each histogram chart represents the decrease in DiOC6(3) labeling after incubation with the medium only, As_2_O_3_ (Ctrl+), rMIC1, or rMIC4. The bar graph representing the histogram results is shown on the right side of the figure. (**C**) Chromatin fragmentation was assessed after treating Jurkat cells (5 × 10^6^ cells/mL) with 5 µg/mL rMIC1, rMIC4, or 10 µM As_2_O_3_ (positive control) for 16 h. Genomic DNA was extracted and analyzed using a 1.5% TAE agarose gel. Lane M: molecular size marker (DNA ladder), Lane 1: negative control (PBS-treated cells); Lane 2: positive control (As_2_O_3_-treated cells), Lanes 3 and 4: rMIC1- and rMIC4-treated cells, respectively. Results are mean ± SD of triplicates from one representative experiment repeated three times with similar outcomes. * *p* < 0.05 compared to control groups.

The effects observed after lectin stimulation of cells are often due to cross-linking and stimulation of various cell receptors, leading to the activation of multiple signaling cascades, resulting in either cell proliferation or cell death [29]. Therefore, we treated Jurkat cell cultures with varying concentrations of rMIC1 or rMIC4 for 24 h and analyzed apoptosis by labeling annexin V-FITC and PI. We observed that the lectins induced apoptosis by increasing the proportion of positive cells for annexin V compared to unstimulated cells in a dose-dependent manner (Figure 5, panel A). To confirm cell death, we incubated the cells with DiOC6(3), which is retained by the mitochondria of healthy cells. During apoptosis, cells lose the ability to maintain mitochondrial potential and fail to retain DiOC6(3). After 24 h of stimulation with rMIC1 and rMIC4, we observed a significant loss in mitochondrial membrane potential (Figure 5, panel B). Furthermore, we observed increased DNA fragmentation in Jurkat cells stimulated with rMIC1 and rMIC4, a typical characteristic of apoptotic cells, compared to unstimulated cells (Figure 5, panel C). These data suggest that MIC1 and MIC4 induce apoptosis in Jurkat cells.

**Figure 6:**
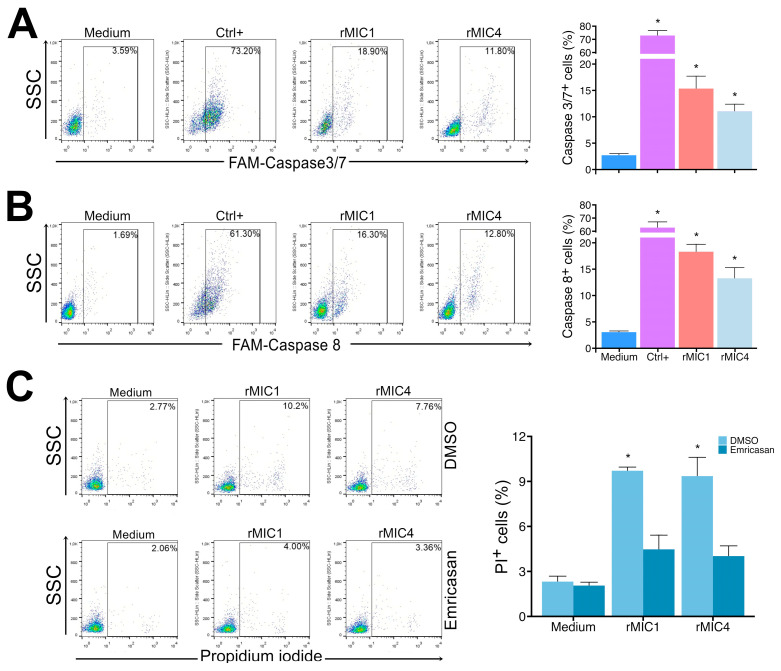
rMIC1 and rMIC4 increase caspase-3/7 and caspase-8 activities in Jurkat cells. (**A**) Jurkat cells (2.5 × 10^5^ cells/mL) were treated with 5 µg/mL rMIC1 or rMIC4 for 24 h and labeled for caspase-3/7 activity. (**B**) Caspase-8 activity was assessed in the same way. (**C**) rMIC1 or rMIC4-stimulated Jurkat cells were incubated in the presence of DMSO (vehicle) or 20 μM pan-caspase inhibitor Emricasan for 1 h, stained with PI. Samples were analyzed by cytometry. The bar graphs representing the dot blot results are shown on the right side of the figure. Results are mean ± SD of triplicates from a representative experiment repeated three times with similar outcomes. * *p* < 0.05 compared to the unstimulated cell group (medium).

To characterize whether rMIC1 and rMIC4 induced apoptosis in Jurkat cells through an extrinsic pathway, we labeled active caspase-3/7 and caspase-8 in rMIC1- or rMIC4-stimulated Jurkat cells. As shown in Figure 6, rMIC1 and rMIC4 led to a significant increase in active caspase-3/7 levels (positive cells) compared to unstimulated cells. When we analyzed active caspase-8, we also detected a significant increase 24 h after stimulating Jurkat cells with rMIC1 or rMIC4 compared to unstimulated cells. Moreover, when we used the pan-caspase inhibitor Emricasan in rMIC1- and rMIC4-stimulated Jurkat cells, we observed no difference in the relative number of PI-positive cells compared to unstimulated cells (Figure 6, panel C). Our results indicate that the apoptosis of Jurkat cells induced by rMIC1 and rMIC4 depends on the caspase activation.

**Figure 7:**
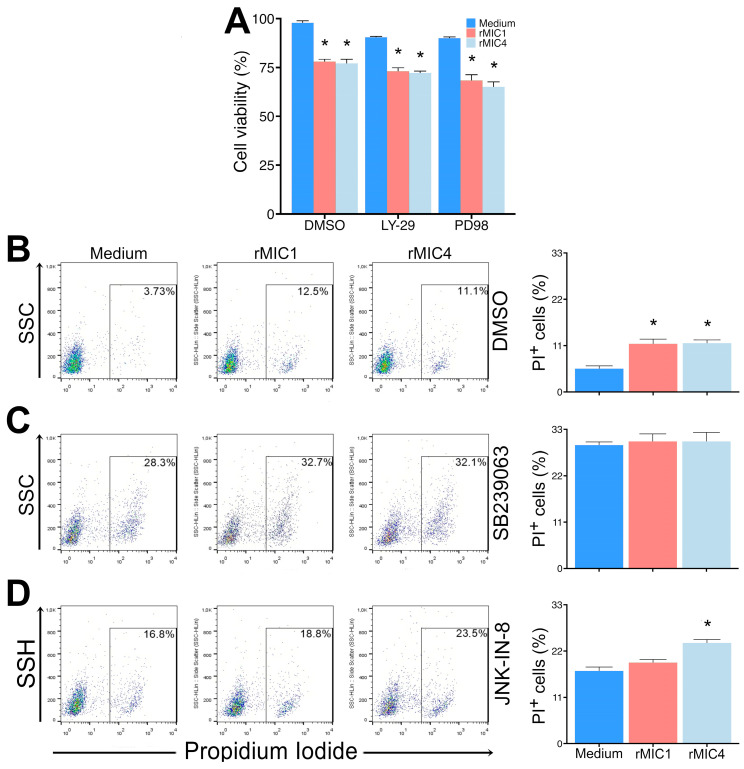
rMIC1 and rMIC4 activate MAPK pathways to induce Jurkat cell death. Jurkat cells (2 × 10^5^ cells/mL) were pre-treated for 1 h with one of the following inhibitors or control treatments: (**A**) LY-29 (a PI3K inhibitor), PD98059 (PD98, an ERK inhibitor), (**B**) non-stimulated (medium) and DMSO-treated cells as controls, (**C**) SB239063 (p38 inhibitor), and (**D**) JNK-IN-8 (JNK inhibitor). After pre-treatment, cells were stimulated for 24 h with 5 µg/mL rMIC1 or rMIC4. Mitochondrial activity was assessed by MTT reduction, as described in the legend in Figure 3A, while PI-positive cells were detected with cytometry. Results, expressed as mean ± SD of triplicates from a representative experiment repeated three times with similar outcomes, are shown in histograms representing one triplicate. * *p* < 0.05 compared to the negative control. Results are presented as mean ± SD and represent three independent experiments.

Cells can respond to stress through various mechanisms that rely on the activation of highly conserved protein kinases, such as MAPK and PI3K/Akt. These signaling pathways seem implicated in cell survival and death [30,31]. To characterize the rMIC1- and rMIC4-induced apoptosis in Jurkat cells, we stimulated cells with pharmacological inhibitors that block apoptotic signaling pathways. Apoptosis in Jurkat cells stimulated with rMIC1 and rMIC4 was not affected when we used PD9805, an ERK inhibitor, or LY294002, a PI3K inhibitor (Figure 7, panel A). Unlike the control (Figure 7, panel B), which showed increased cell death even in the presence of the vehicle, the p38 MAPK inhibitor (SB239063) led to a decrease in apoptosis of rMIC1- or rMIC4-stimulated Jurkat cells to the levels of unstimulated cells (Figure 7, panel C). In turn, the JNK MAPK inhibitor (JNK-IN-8) decreased cell apoptosis only in cells stimulated with rMIC1 but not with rMIC4 (Figure 7, panel D). However, we observed increased PI staining in cells treated with SB239063 or JNK-IN-8 alone, indicating higher rates of necrosis. This suggests that, while these inhibitors modulate apoptosis, their inhibition also compromises Jurkat cell survival, which is consistent with the expected increase in mortality observed for MIC4 in experiment 7D. Our data suggest that rMIC1 activates P38 and JNK MAPK signaling to trigger cell death in Jurkat cells, whereas rMIC4 activates P38 MAPK but not JNK MAPK. Furthermore, MAPK/ERK and PI3K/AKT do not appear to participate in rMIC1- and rMIC4-induced apoptosis signaling in Jurkat cells.

### 3.4. Release of ROS from the NADPH Oxidase Complex Is Responsible for Apoptosis of Jurkat Cells Induced by rMIC1 or rMIC4

**Figure 8:**
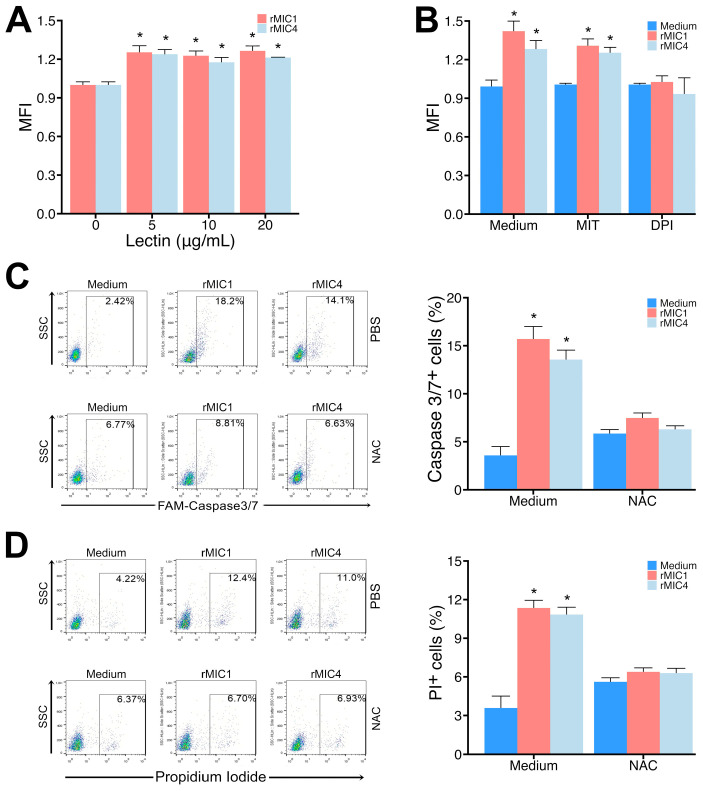
Increased caspase-3 activity in Jurkat cells stimulated with rMIC1 or rMIC4 is dependent on ROS release mediated by NADPH oxidase. (**A**) Jurkat cells (2 × 10^5^ cells/mL) were stained with 5 μM DCFDA and stimulated with varying rMIC1 or rMIC4 concentrations for 2 h. (**B**) To identify the source of ROS, the cells were incubated with mito-TEMPO (MIT) at 20 μM or diphenylene iodonium (DPI) at 20 μM for 45 min before stimulation with 5 µg/mL of rMIC1 or rMIC4 for 2 h. The culture plates in panels (**A**,**B**) were read using a fluorescent microplate reader. (**C**,**D**) Cells were stimulated with 5 µg/mL rMIC1 or rMIC4 and treated with 1 mM N-acetyl-cysteine (NAC). After 24 h, the cells were labeled with FAM-Caspase-3/7 (panel (**C**)) or PI (panel (**D**)) and analyzed by cytometry. Results, expressed as mean ± SD of triplicates from a representative experiment repeated three times with similar outcomes, are shown in histograms representing one triplicate. * *p* < 0.05 compared to the unstimulated cell group (0 or medium).

The p38 and JNK MAPK pathways are involved in the cellular stress response, inducing ROS production, as shown after incubating cells with different lectins [32,33,34,35]. However, we found no reference in the literature regarding increased mitochondrial ROS production after culture with *T. gondii* microneme proteins. Thus, we evaluated whether Jurkat cells would increase ROS production after stimulation with rMIC1 or rMIC4. Our data indicate that a 2-h stimulation of Jurkat cells with rMIC1 or rMIC4 increased ROS detection compared to unstimulated cells (Figure 8, panel A). Next, we evaluated the source of ROS production by using an inhibitor of the NADPH oxidase complex (DPI) and a reducing agent preferentially sequestered in the mitochondria (MIT). Our data suggest that DPI, but not MIT, was able to block ROS release (Figure 8, panel B). We then evaluated whether adding the ROS scavenger NAC to the Jurkat cell cultures would reduce the apoptotic effect caused by the lectins, as detected by a decrease in the caspase-3/7- and PI-positive cells. Our results showed that Jurkat cells stimulated with rMIC1 or rMIC4 in the presence of reducing agents had diminished caspase-3/7 activation and PI labeling (Figure 8, panels C and D, respectively). Together, these data demonstrate that ROS production leads to caspase activation, which triggers apoptosis in Jurkat cells.

## 4. Discussion

The current study demonstrated that the recombinant microneme proteins MIC1 and MIC4 interact with Jurkat cells, a human T-cell leukemia line, inducing programmed cell death by apoptosis in a carbohydrate recognition-dependent manner. This apoptosis was driven by increased ROS production, which subsequently activated MAPK signaling pathways. In addition, Jurkat cells incubated with rMIC1 or rMIC4 exhibited elevated levels of activated caspase-8, implicating both intrinsic (mitochondrial) and extrinsic (death receptor) apoptotic pathways.

Lectins use diverse pathways to induce apoptosis, making them versatile agents for targeting cancer cells. The activation of both caspase-dependent and mitochondrial pathways enhances the potential of lectins to overcome resistance mechanisms in cancer cells, which are frequently designed to evade apoptosis through single-pathway suppression [36,37,38]. By engaging intrinsic and extrinsic pathways, lectins such as MIC1, MIC4, and others might increase the robustness of apoptosis induction, which is critical for effective cancer therapy.

Previous studies have highlighted the multifaceted roles of lectins in inducing apoptosis in cancer and immune cell models [25,37,39,40,41,42]. Some research has focused on the distinct apoptotic pathways in which lectins activate in Jurkat cells, with particular attention to plant lectins such as ArtinM, a D-mannose-binding jacalin-related lectin from *Artocarpus heterophyllus* seeds, which activates T cells and induces cell death in Jurkat cells by interacting with specific glycan structures on their surface [39]. The dual functionality of lectins as modulators of immune responses and inducers of programmed cell death implies their potential for therapeutic applications in the selective targeting of cancer cells [27]. Regarding ArtinM, its ability to trigger apoptotic pathways in cancer cells without affecting healthy cells illustrates its promising potential as a targeted anticancer agent [39].

The properties of ArtinM appear to be shared by two other jacalin-related lectins from *Morus nigra*, Morniga-M and Morniga-G. Interestingly, Morniga-M, a mannose-specific lectin, has been described as activating T cells and is toxic for Jurkat T cells [43]. Morniga-G, a T/Tn-specific lectin from *Morus nigra*, binds to the DR5 receptor, a death receptor involved in extrinsic apoptosis, activating caspase-8 and initiating a caspase cascade that culminates in cell death. Additionally, Morniga-G disrupts mitochondrial function, a hallmark of the intrinsic apoptotic pathway, thereby triggering this pathway as well [44]. Our findings align with this dual activation model, as rMIC1 and rMIC4 not only increased caspase-8 activity but also reduced mitochondrial potential in Jurkat cells, suggesting that MIC1 and MIC4 engage both apoptotic pathways to induce cell death effectively.

A significant implication for cancer therapy is the selectivity of lectins for cancer cells. Pujari et al. [45] demonstrated that the lectin derived from the fungus *Rhizoctonia bataticola* (RBL) induces apoptosis in Jurkat cells primarily through the extrinsic pathway involving caspase-8. They showed that RBL acted selectively for leukemic cells, sparing normal CD3^+^ T cells and CD34^+^ progenitor cells, thereby indicating its potential as a selective therapeutic agent. Similarly, Perišić Nanut et al. [41] showed that the lectin from the fungus *Clitocybe nebularis* (CNL) selectively binds to glycoprotein receptors on Jurkat cells, leading to caspase activation and apoptosis. This selectivity may be due to unique glycan structures on leukemic cells, allowing lectins to act as molecular keys to trigger cell death in malignant cells while minimizing off-target effects. These findings suggest that lectins like RBL, CNL, MIC1, and MIC4 could serve as effective anticancer agents by targeting specific carbohydrate structures that are overexpressed or uniquely configured in cancer cells.

The ability to induce apoptosis is also present in animal lectins, such as galectins and leczyme. Galectins are a family of evolutionarily conserved soluble proteins that bind to β-galactosides and are often associated with inflammation, infection, and tumor development. Galectin-1, for instance, induces apoptosis, particularly in immune cells such as T cells, by binding to cell surface glycoconjugates. This process plays a role in immune regulation, as galectin-1 can limit inflammation by promoting the death of activated T cells. It can also induce apoptosis in cancer cells under certain conditions, though cancer cells often develop mechanisms to resist its effects [45,46]. Galectin-3 can play dual roles in apoptosis depending on its location. Inside the cell, galectin-3 typically acts as an anti-apoptotic molecule, helping cells resist programmed cell death and supporting cancer cell survival. However, when located outside the cell, galectin-3 can interact with surface receptors to promote apoptosis in specific situations [47,48]. Like rMIC1 and rMIC4, galectin-3 influences ROS production and activates intracellular MAPK signaling pathways when inducing apoptosis [49]. Leczyme, a sialic acid-binding lectin from *Rana catesbeiana* oocytes, binds to sialic acid residues on the cell surface, triggering mitochondrial disruption and the subsequent activation of caspases, particularly caspase-3, leading to apoptosis [50]. Our current findings are in accordance with this mechanism. However, we have demonstrated that rMIC1 and rMIC4 activate both the extrinsic and mitochondrial apoptotic pathways in Jurkat cells.

Temperature dependency in lectin–cell interactions has been noted in previous research, adding another layer of selectivity and specificity. Mayerhofer et al. [51] observed that certain lectins require specific temperature ranges to perform optimally. In our study, we found that MIC1 and MIC4 exhibited a slightly stronger interaction with Jurkat cells at 37 °C compared to 4 °C, which may be associated with the temperature dependence of *T. gondii* to infect cells, determining its ability to infect cold- or warm-blooded vertebrates [52]. This temperature dependency could be an essential factor in the practical application of lectins as therapeutic agents, as it may allow for control over lectin binding and apoptotic induction based on environmental conditions.

Given the distinct specificities of MIC1 and MIC4, which primarily bind to glycoconjugates with terminal sialic acid residues and those terminating in galactose, respectively [13,53], we initially hypothesized that they would not compete for the same glycoconjugate binding sites. However, our results revealed an intriguing competitive interaction between MIC1 and MIC4. The addition of unlabeled MIC4 reduced the binding of labeled MIC1 to Jurkat cells, whereas the reverse effect was not observed. These findings suggest that MIC4 has a higher affinity for, or a larger binding footprint on, specific glycan receptors, which leads to steric hindrance. The proximity of these binding sites likely allows MIC4 to inhibit MIC1 binding, providing valuable insights into the structural and spatial requirements for glycan recognition on cell surfaces utilized by *T. gondii*.

Finally, the therapeutic potential of lectins extends beyond cancer therapy, raising the question of whether MIC1 and MIC4 might similarly influence immune cells, as seen in mouse dendritic cells and macrophages that respond to these microneme proteins by producing the pro-inflammatory cytokine IL-12, thus promoting a protective T helper 1 (Th1) immune response against *T. gondii*. The protective potential of MIC1 and MIC4 is evidenced by studies utilizing these proteins as experimental vaccines in mice, demonstrating that vaccinated animals had a significantly reduced brain cyst burden and lower mortality rates compared to unvaccinated controls following infection [20,21].

## 5. Conclusions

Our study contributes to the growing evidence that lectins, such as MIC1 and MIC4, can induce selective apoptosis in leukemic cells through complex, caspase-dependent mechanisms involving extrinsic and intrinsic pathways. Furthermore, it opens up new perspectives for uncovering whether MIC1 and MIC4 act on human immune cells, as observed in mice dendritic cells and macrophages that respond to them by producing the pro-inflammatory cytokine IL-12. This interaction could be relevant to designing a vaccine against toxoplasmosis and exploiting potential immunomodulatory applications.

## Data Availability

Data are contained within the article.

## Supplementary Materials

The following supporting information can be downloaded at https://www.mdpi.com/article/10.3390/pathogens14040372/s1: Figure S1: Gate strategy used to analyze the cytometry data.
