# Supplementary material for: Recombinant Microneme Proteins MIC1 and MIC4 from Toxoplasma gondii Cause Cytotoxic Effects in the Human Jurkat T-Lymphocyte Cell Line
Source: Pathogens. 2025 Apr 9;14(4):372. doi: 10.3390/pathogens14040372 (PMC12030039; doi:10.3390/pathogens14040372)
Supplement: Supplementary file 1 [file pathogens-14-00372-s001.zip › pathogens-3492571-supplementary.pdf]

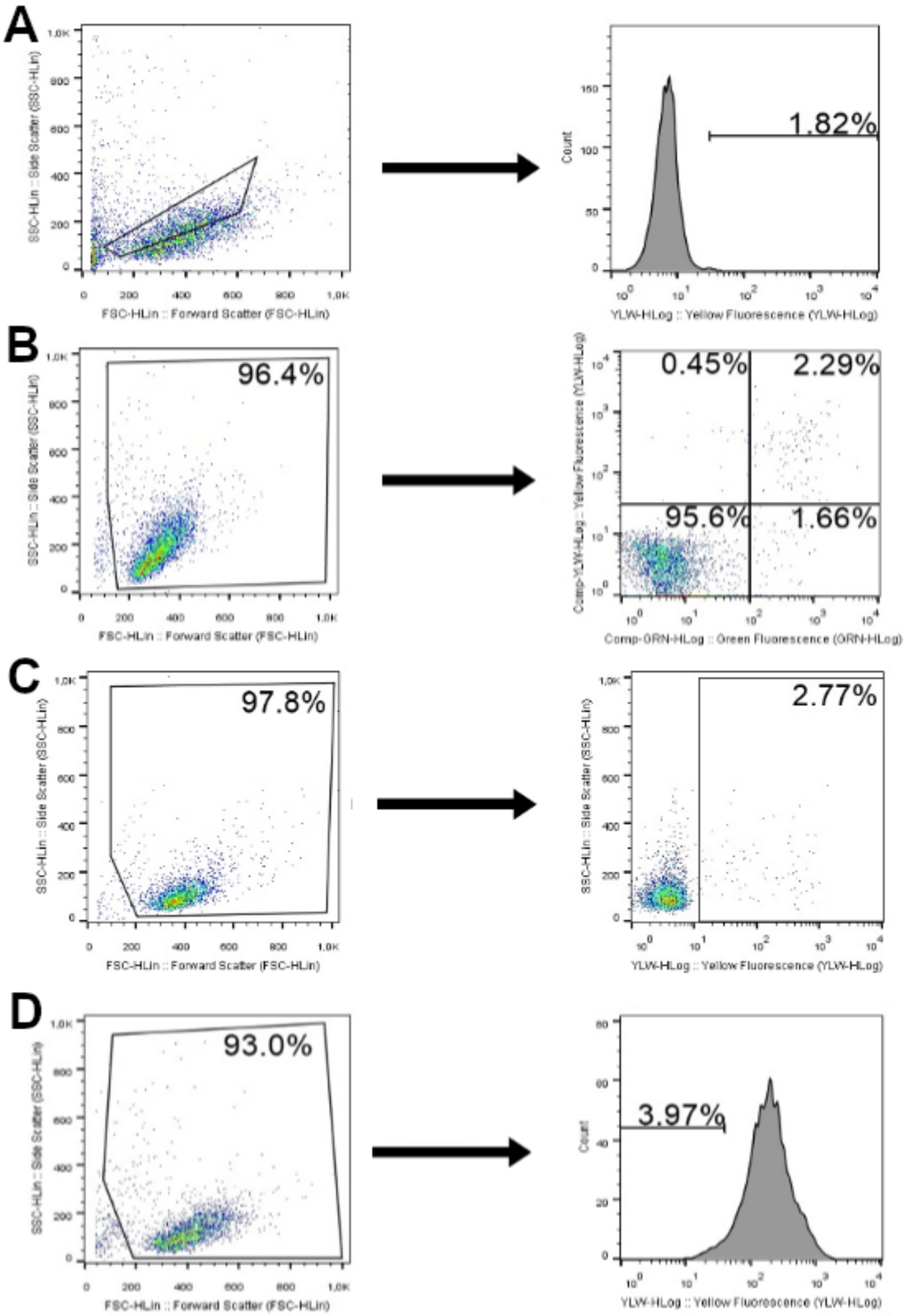

Figure S1: Gate strategy used to analyze the cytometry data.

A- The gate was used to separate doublets. The histogram shows the binding levels of biotinylated rMIC1 or rMIC4, detected using streptavidin-FITC labeling.

B- Illustrates the gating strategy for cells labeled with Annexin-FITC and propidium iodide.

C- Gating strategy used to analyze cytometry data for caspase-8-FAM, caspase-3/7-FAM, and propidium iodide.

D- The histogram shows the mitochondrial membrane potential measured using DIOC6(3) dye.
